# Optimising Paracetamol Prescribing for Safer, Greener, and Cost-Effective Care: Lessons From an Ongoing Emergency Department Quality Improvement Project

**DOI:** 10.7759/cureus.92108

**Published:** 2025-09-11

**Authors:** Vijay Lakshmanan, Michael Rosser

**Affiliations:** 1 Hospital Medicine, Lancashire Teaching Hospitals NHS Foundation Trust, Preston, GBR; 2 Emergency Medicine, Lancashire Teaching Hospitals NHS Foundation Trust, Preston, GBR

**Keywords:** emergency departments, environmental sustainability, healthcare sustainability, hospital sustainability, intravenous paracetamol, low-cost healthcare, paracetamol, quality improvement project, simple quality improvement project, sustainability in healthcare

## Abstract

Paracetamol is one of the most frequently prescribed analgesics in emergency departments (EDs). While intravenous (IV) and oral formulations provide similar analgesic effects, IV paracetamol is more expensive and has a carbon footprint several-fold higher than oral administration. In patients who can take oral medication, the oral route is safer, greener, and more cost-effective. At Royal Preston Hospital in Preston, UK, the baseline audit data from December 2022 to November 2023 revealed that IV paracetamol accounted for approximately 38% of all 1 g paracetamol doses prescribed in the ED. Following informal teaching sessions and staff engagement, a re-audit from February to April 2024 demonstrated no significant reduction in IV use. A further audit from May 2024 to March 2025 revealed no significant reduction in IV paracetamol use, with prescribing rates continuing to hover around 35% to 40% across most months.

These findings highlight that early educational interventions can influence prescribing behavior but may be insufficient to deliver lasting change. To build on this work, a structured quality improvement (QI) approach was adopted to implement more targeted and sustainable interventions, including formal teaching, visual prompts, and regular feedback cycles. By encouraging appropriate use of oral paracetamol, this project aims to reduce unnecessary IV prescribing while improving patient safety, lowering costs, and contributing to environmentally sustainable practice.

Importantly, this remains an ongoing, multi-cycle initiative, with a third round of data collection commencing in August 2025. This QI report aims not only to outline the effectiveness and sustainability impact of optimizing paracetamol prescribing but also to demonstrate how similar projects can be replicated across various other healthcare departments worldwide. Such initiatives have the potential to promote safer, greener, and more cost-effective care with global relevance.

## Introduction

Paracetamol is one of the most commonly used analgesics in emergency departments (EDs), valued for its effectiveness and generally favourable safety profile. However, prescribing practices do not always reflect cost or environmental considerations, with intravenous (IV) paracetamol frequently administered despite offering no greater pain relief than the oral route, while being more expensive and associated with a significantly higher carbon footprint [1]. 

A multicentre life-cycle assessment found that producing and administering a 1 g oral paracetamol tablet generates about 38 grams of carbon dioxide (CO₂) equivalent (CO₂e, a standard measure that expresses all greenhouse gases in terms of their warming effect compared to CO₂). In contrast, a single 1 g IV dose results in 310-628 grams of CO₂e depending on packaging, making IV administration up to 16 times more carbon intensive than the oral route [2]. To put this into perspective, giving one IV dose instead of an oral tablet creates a carbon footprint comparable to driving a typical petrol car for one to two miles, whereas the oral dose is closer to just a few hundred metres. 

Financially, the disparity is just as significant. According to the NHS Drug Tariff issue (December 2019), a 1 g oral paracetamol tablet costs approximately £0.03 compared with £1.20 for a 1 g intravenous vial, making IV administration nearly 40 times more expensive [3].

Despite these differences, many hospitals continue to favour IV use, often due to default habits or misconceptions about efficacy. However, evidence supports that when oral intake is feasible, switching to the oral route avoids unnecessary costs, reduces environmental impact, and does not compromise analgesic efficacy [1].

At Royal Preston Hospital ED, Preston, UK, our baseline audit data from December 2022 to November 2023 showed that IV paracetamol accounted for approximately 38% of all 1 g doses prescribed. Following informal teaching presentations and staff engagement, a re-audit (February 2024 to April 2024) demonstrated no significant improvement. A subsequent audit (December 2024 to March 2025) again showed little meaningful change, reinforcing the need for more structured, strategic interventions to achieve durable and sustainable improvement.

Accordingly, we initiated a structured quality improvement (QI) project with the aim of reducing IV paracetamol use. The objective of this project was to reduce the proportion of IV paracetamol prescribed in the ED by at least 10% by the end of 2025, with the overarching goals of improving patient safety, reducing unnecessary costs, and lowering environmental impact through more sustainable prescribing practices.

Interventions included educational posters, formal doctor and nurse teaching sessions, and awareness emails, with the caveat that IV use must remain appropriate for patients who cannot tolerate oral medication.

Through this QI initiative, our goal is not only to enhance patient safety and cost-efficiency but also to contribute to environmentally sustainable prescribing practices. This report outlines how proper QI methodology was used to design a multi-cycle project, enabling interventions to be implemented and re-audited so that change can be monitored and refined over time. Beyond our local department, it also serves to demonstrate how a simple project of this nature can have broader relevance from both cost and sustainability perspectives, emphasising that the aim is not only to present results but also to showcase an ongoing project with wider applicability.

## Materials and methods

Baseline data were collected retrospectively from pharmacy dispensing records to capture the total number of 1 g oral and IV paracetamol doses issued to the Royal Preston Hospital ED. To obtain these figures, we liaised directly with hospital pharmacists and obtained access to pharmacy dispensary records, which reported the number of packs of oral paracetamol (100 tablets per pack) and IV paracetamol (10 vials per pack) supplied to the department on a monthly basis. Using simple calculations, we converted these values into the total number of 1 g oral and IV issues per month. This analysis demonstrated that a significant proportion of paracetamol was being administered intravenously despite the availability of the oral route.

In response, informal interventions such as ad hoc teaching sessions, electronic posters, and word-of-mouth staff engagement were trialled. A subsequent cycle of data collection following these efforts showed negligible improvement in prescribing practice, indicating that informal efforts alone were insufficient to achieve lasting change.

To address this, we decided to adopt a structured QI approach using the Model for Improvement. This framework is built around three guiding questions: defining a clear aim, establishing measures to track progress, and identifying changes likely to lead to improvement, combined with iterative Plan-Do-Study-Act (PDSA) cycles to test and refine interventions [4].

In line with this, a Specific, Measurable, Achievable, Relevant, Time-Bound (SMART) aim was defined: to reduce the proportion of IV paracetamol prescribed in the ED by at least 10% by the end of 2025, a threshold chosen as a pragmatic and achievable benchmark based on comparable local QI projects. Progress will be tracked using monthly pharmacy dispensing data, while targeted educational and awareness interventions such as posters, teaching sessions, and audit feedback represent the planned changes to drive improvement. Importantly, IV use will continue to be encouraged when clinically appropriate.

**Figure 1:**
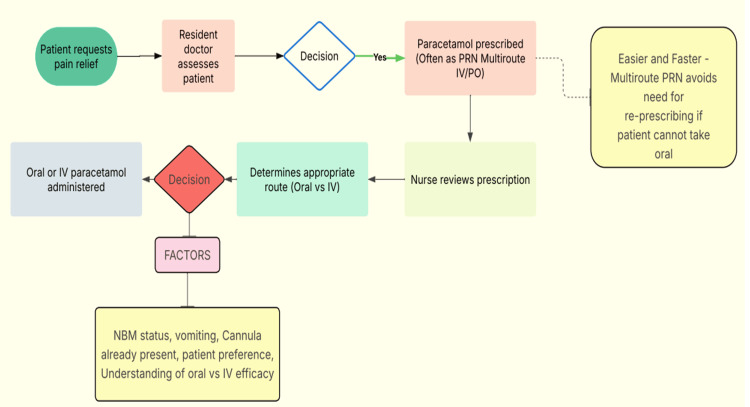
Process map of the paracetamol prescribing pathway in Royal Preston Hospital ED This process map illustrates the step-by-step pathway by which a patient in the ED may receive either oral or IV paracetamol. By breaking the pathway down in this way, key decision points become clearer, allowing us to identify where issues may arise and to design targeted interventions to address them. PRN: Pro re nata (as needed); PO: per oral (by mouth); IV: intravenous; NBM: nil by mouth

As part of this structured approach, a process map was created to illustrate the typical prescribing pathway and highlight key decision points contributing to IV use, such as IV cannulae already being in place, assumptions about faster onset, and misinterpretation of nil-by-mouth criteria (Figure 1). These insights were informed by informal staff surveys and direct observations from clinical practice within the ED and directly shaped targeted interventions: Posters highlighted the equivalent onset and efficacy of oral paracetamol, teaching sessions clarified nil-by-mouth criteria, and a structured handover message was introduced to encourage ordering oral paracetamol rather than multi-route prescriptions to avoid unnecessary IV use.

**Figure 2:**
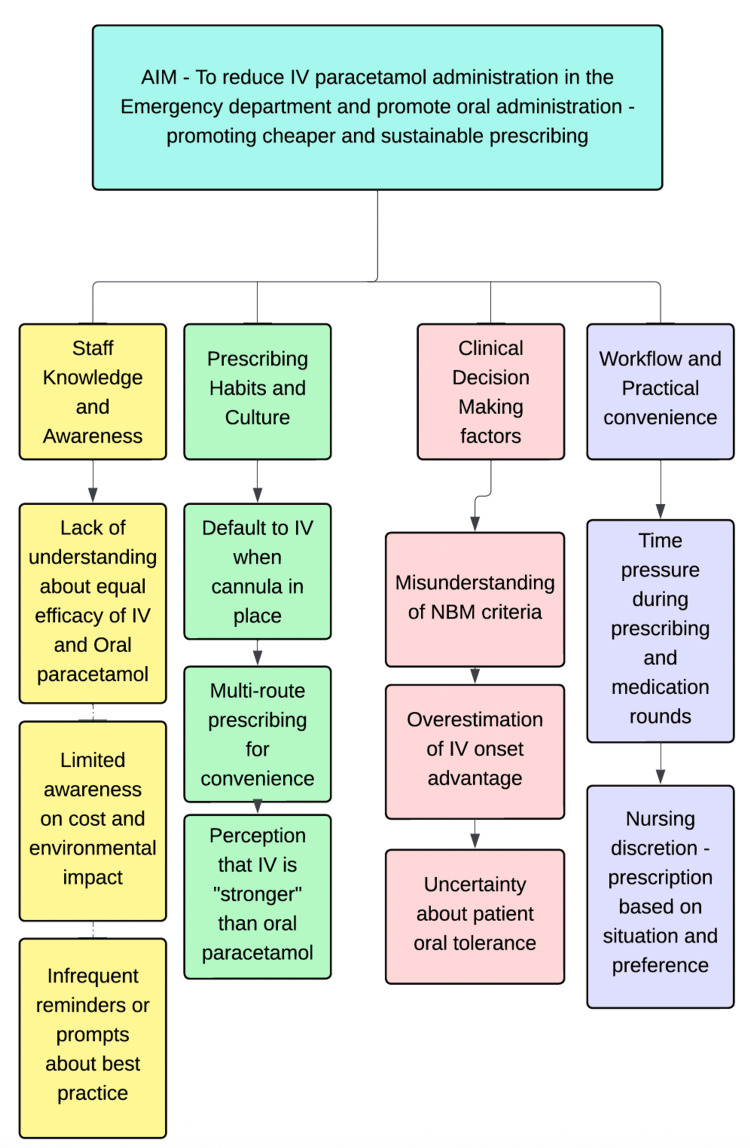
Key drivers influencing IV paracetamol prescribing in the ED Information was derived from insights gathered through an informal staff survey. IV: intravenous

A driver diagram was also developed to identify the primary factors influencing prescribing practice, including staff knowledge and awareness, habitual prescribing patterns, clinical considerations, and workflow-related convenience. This analysis informed the design of targeted interventions (Figure 2).

**Figure 3:**
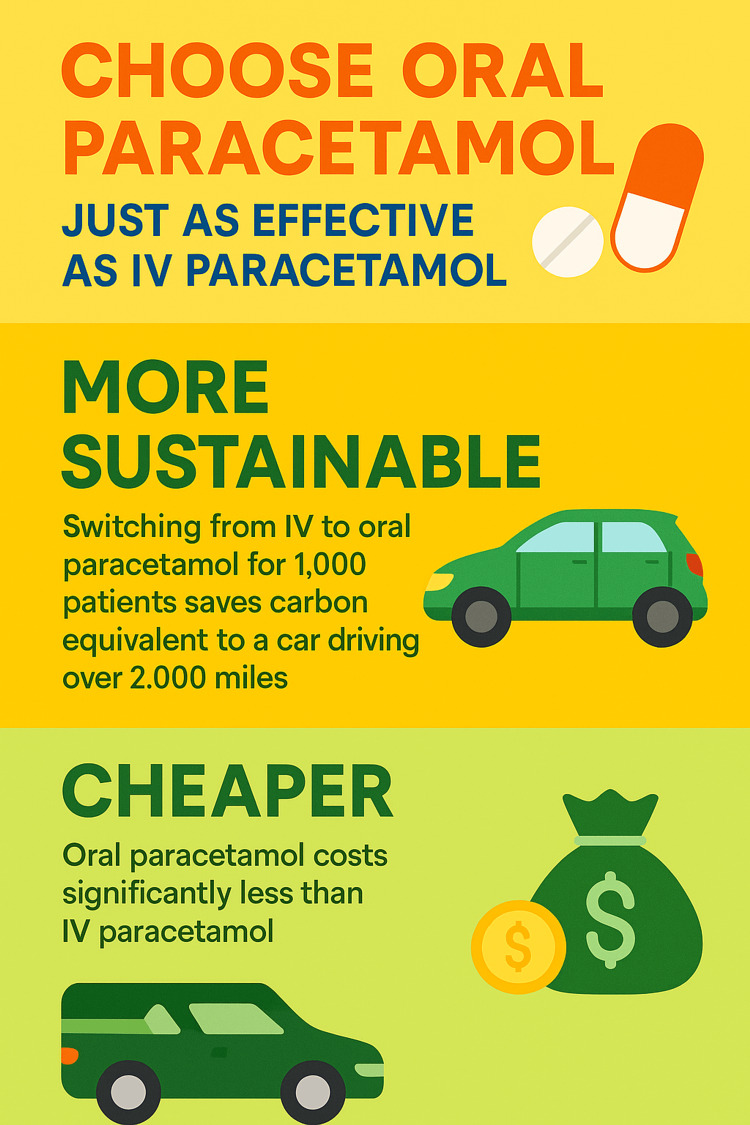
Educational poster used as part of the intervention strategy The poster was displayed in staff areas to provide a visual and continuous reminder of the cost, sustainability, and clinical considerations when choosing between oral and IV paracetamol. IV: intravenous

Building on these insights, the project advanced into a third cycle with the introduction of more strategic interventions. These included: 1. An educational poster was displayed in common staff areas to provide a continuous visual reminder of cost, sustainability, and clinical considerations when prescribing paracetamol (Figure 3); 2. A department-wide email bulletin summarising the cost and sustainability differences between IV and oral paracetamol; 3. Targeted teaching sessions for junior doctors and nursing staff which are planned to be short and concise. They will briefly highlight research demonstrating the similar efficacy of oral and IV paracetamol, outline the cost and sustainability impact, and present our audit results. The sessions will aim to encourage doctors to prescribe oral paracetamol where appropriate (instead of multi-route) and nurses to prioritise oral administration. 4. A presentation at the ED audit meeting to raise awareness and encourage senior support. Following this meeting, it was agreed that junior doctors should be advised to prescribe oral-only paracetamol instead of multiroute (whenever clinically appropriate) in order to reduce unnecessary administration of IV formulations. 5. A short message read out during daily handover meetings, encouraging doctors to prescribe oral-only paracetamol rather than defaulting to multi-route prescribing.

All of the above interventions were implemented during August 2025. At the time of submission, the targeted teaching sessions had not yet been completed, but are scheduled to begin in September 2025, with at least one doctor-focused session and one nursing staff session planned each month. Each session will be short, lasting approximately 10 to 15 minutes, to ensure feasibility within busy departmental schedules.

**Figure 4:**
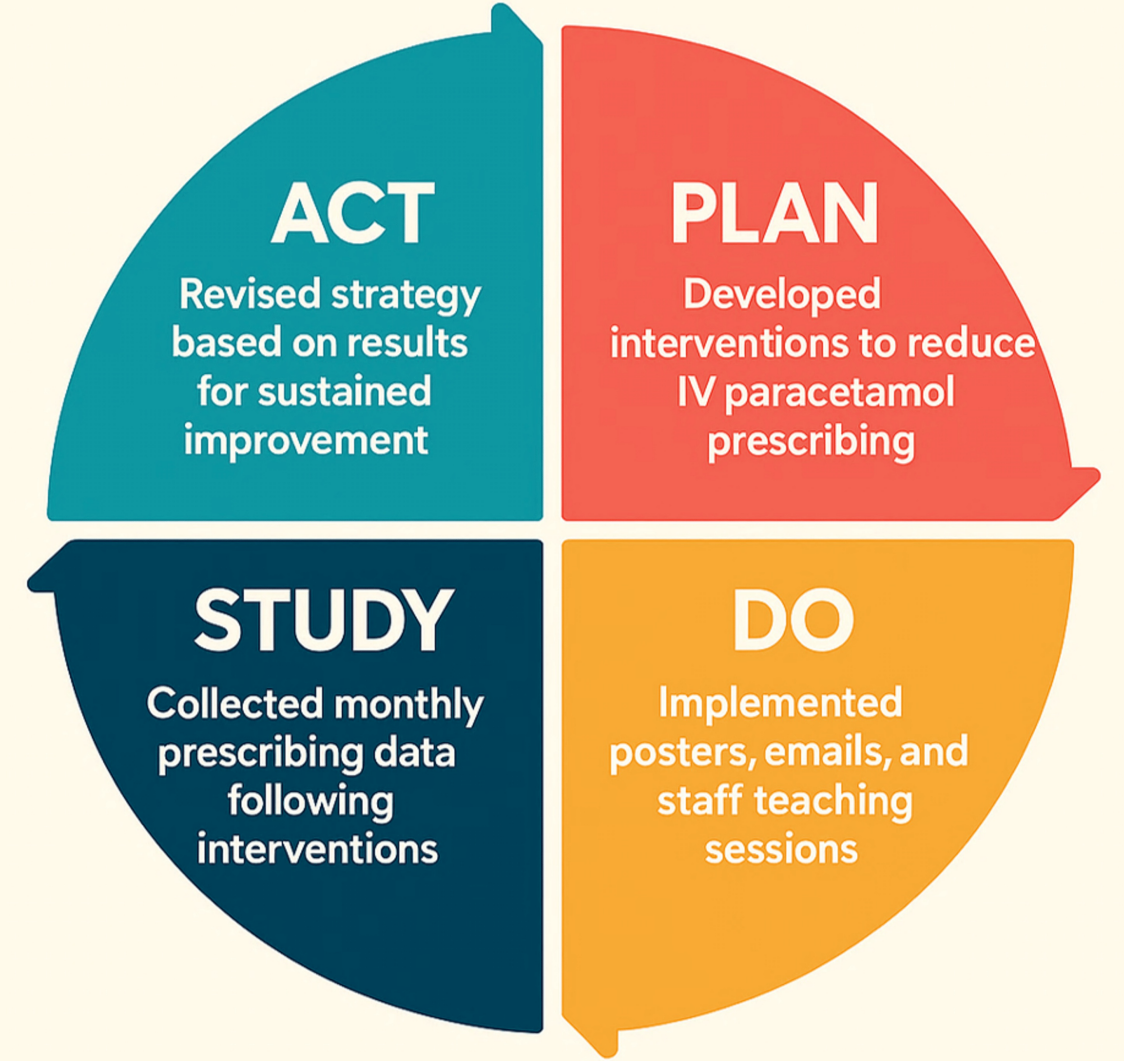
Plan-Do-Study-Act (PDSA) cycle applied to optimise paracetamol prescribing This diagram illustrates the iterative quality improvement methodology used in the project. Each cycle tests targeted interventions, analyses their impact, and refines strategies for subsequent cycles. By repeating this structured approach, sustainable change can be achieved while ensuring interventions remain responsive to local needs. IV: intravenous

Data collection for this cycle commenced in August 2025 and will continue through to October 2025, with post-intervention analysis planned for October to December 2025. The project is being delivered through iterative PDSA cycles, with the intention of keeping it ongoing. The overall structure of the PDSA cycle, outlining the stages of planning, implementation, evaluation, and refinement, is illustrated in Figure 4.

Each cycle will refine interventions based on data gathered, with the overarching goal of embedding safer, greener, and more cost-effective prescribing practices within the ED.

## Results

**Figure 5:**
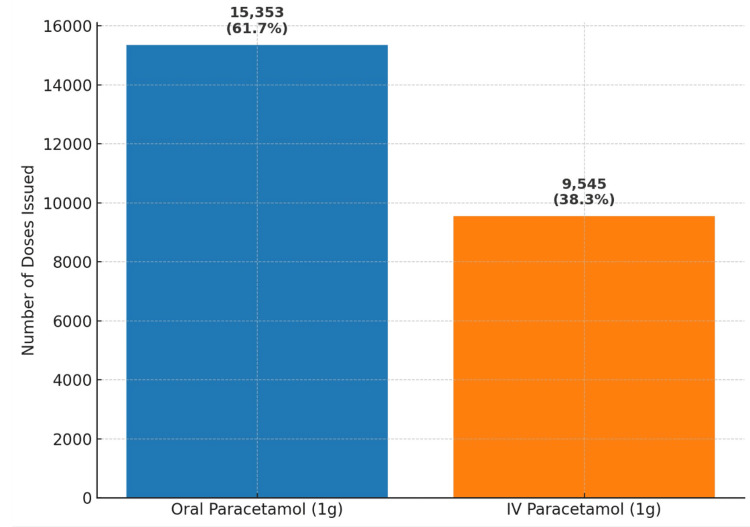
Total number of oral vs. IV paracetamol doses issued in the Royal Preston Hospital ED (December 2022 to November 2023) This bar graph illustrates the baseline distribution of 1 g oral and IV paracetamol doses prescribed in the ED between December 2022 and November 2023. A total of 15,353 oral doses (62%) and 9,545 IV doses (38%) were issued, providing a reference point for subsequent intervention cycles. IV: intravenous

Baseline data were gathered from pharmacy records for the period December 2022 to November 2023 to establish a comprehensive understanding of paracetamol prescribing practice within the ED. This analysis demonstrated that a substantial proportion of paracetamol (approximately 40%) was being administered intravenously despite the availability of the oral route. These findings are illustrated in Figure 5, which presents a bar graph of oral versus IV paracetamol use between December 2022 and November 2023.

**Figure 6:**
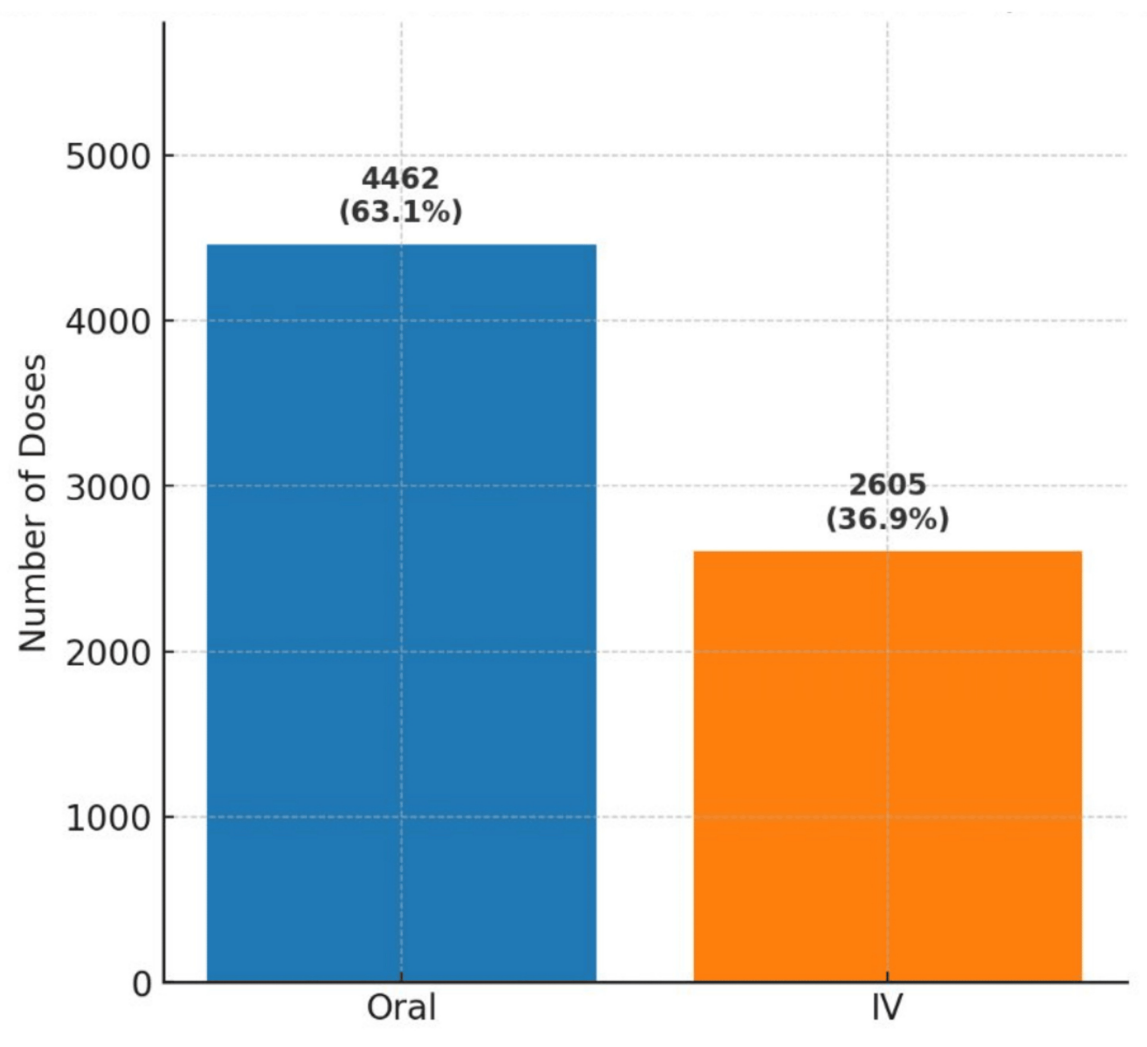
Oral vs. IV paracetamol doses issued in the Royal Preston Hospital ED (February to April 2024) Between February and April 2024, a total of 4,462 oral 1 g doses and 2,605 IV 1 g doses of paracetamol were dispensed to the ED. This equates to an average of 1,487 oral doses (63%) and 868 IV doses (37%) per month. Compared to the baseline data from December 2022 to November 2023, this represents a negligible change, with IV prescribing remaining within the 35% to 40% range. IV: intravenous

In response, informal interventions were introduced, including regular ad hoc teaching presentations, electronic posters, and word-of-mouth staff engagement. A post-intervention audit conducted between February and April 2024 showed a negligible reduction of just around 1.5% in IV paracetamol prescribing. This is demonstrated in Figure 6, a bar graph comparing oral and IV paracetamol use between February and April 2024, which highlights the negligible change following these informal interventions.

**Figure 7:**
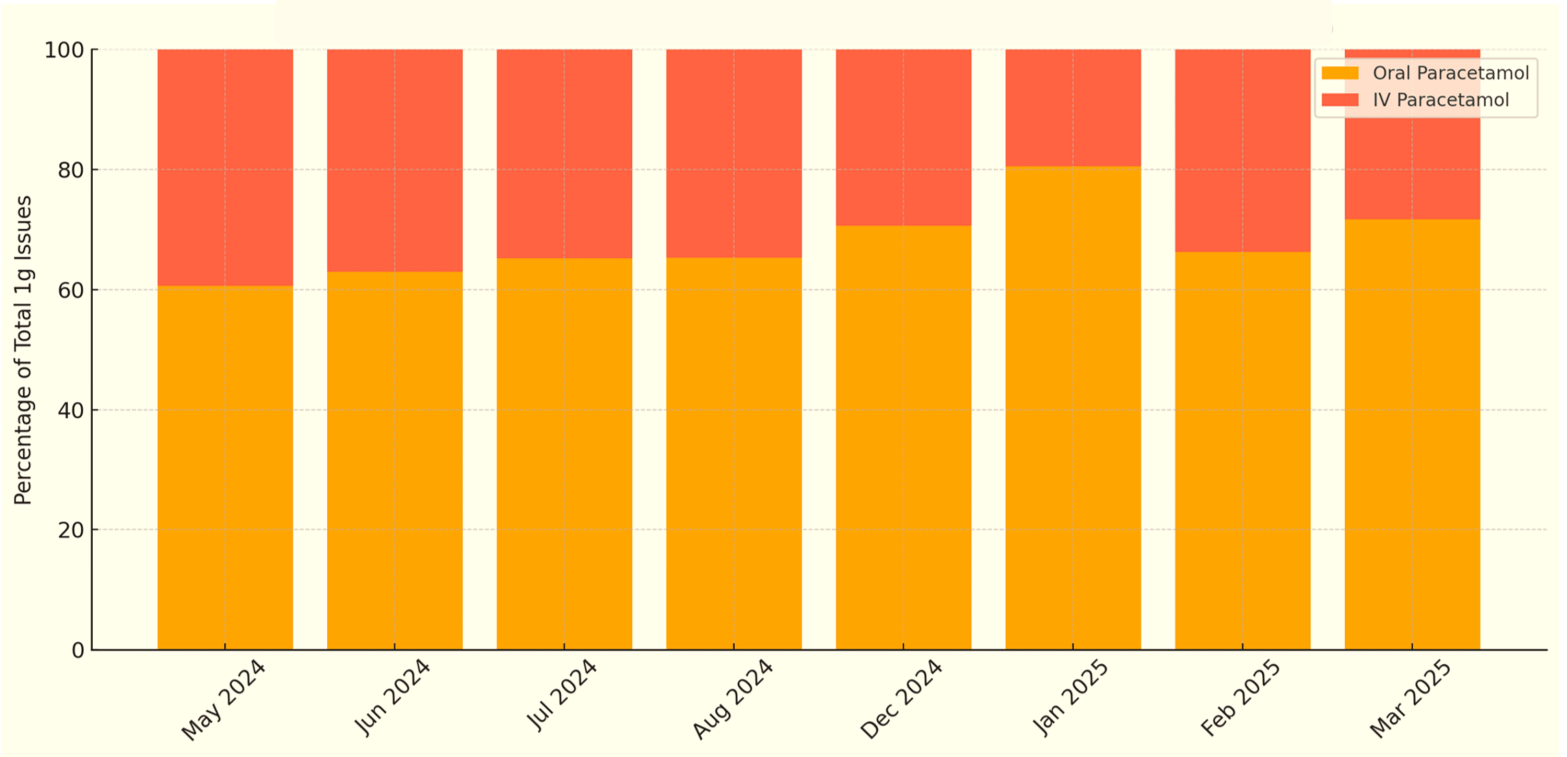
Monthly proportions of oral vs. IV paracetamol prescriptions in Royal Preston Hospital ED (May 2024 to March 2025) Across this period, IV paracetamol use consistently hovered between 35% and 40% of total doses, showing no significant reduction compared with baseline data. These findings highlight the limited impact of informal interventions and the need for a more structured quality improvement approach to drive meaningful change. IV: intravenous

To assess whether these changes occurred in the long term, a further cycle of data collection was performed from May 2024 to March 2025. This showed that while oral prescribing continued to account for the majority of paracetamol use, IV use remained common with no notable improvement from the past. This is shown in Figure 7, which illustrates the monthly percentage proportion of IV versus oral paracetamol use from May 2024 to March 2025.

These findings highlighted that informal measures alone were insufficient to deliver durable change. As such, the project has progressed to a third cycle of structured, strategic interventions, including educational posters, departmental emails, targeted teaching sessions, and formal audit meeting presentations. Data collection for this cycle commenced in August 2025 and will continue until October 2025, with results expected to inform further cycles of improvement.

While measurable impact from these structured interventions has not yet been demonstrated, the aim of this submission is to showcase the application of QI methodology, emphasise the limitations of informal approaches, and continue with iterative cycles to embed sustainable change over time.

## Discussion

Insights from our department and broader relevance

Our QI initiative highlights how optimising the route of paracetamol administration in the ED can influence cost, sustainability, and patient safety, while also demonstrating the challenges of achieving durable behavioural change.

Data Analysis

The data collected through 2023 established that IV paracetamol accounted for approximately 38% of all 1 g paracetamol doses prescribed in the ED. Following informal interventions (including ad hoc teaching, electronic posters, and word-of-mouth reminders), the proportion of IV use between February 2024 and March 2025 remained essentially unchanged, hovering around 35% to 40% across most months, with only minor fluctuations and a small outlier in January. This lack of significant improvement compared with baseline data reinforced the limitations of unstructured, informal approaches. Overall, these results suggested that without consistent reinforcement, prescribing habits tend to revert to familiar patterns.

Importance of Structured Interventions

In contrast to informal approaches, adopting a structured QI framework offers the opportunity to build more sustainable change. By using tools such as process mapping, driver diagrams, and iterative PDSA cycles, the project was able to directly identify and address key barriers. For example, process mapping highlighted misconceptions about nil-by-mouth status, while driver diagrams clarified the influence of entrenched prescribing habits. This ensured that interventions were closely aligned with observed challenges. Furthermore, by presenting results back to the department at regular intervals, the project provides staff with frequent reminders, encouraging accountability and reinforcing desired behaviours. This structured approach increases the likelihood of embedding practice change into the routine workflow.

Broader Relevance

This report demonstrates how structured QI methodology can be applied to a common prescribing practice, with broader relevance beyond a single ED. Audits discussed later in this section have also shown high use of IV paracetamol in settings such as ICUs and surgical wards, where oral administration would often be feasible. These findings suggest that interventions targeting route optimisation could yield financial and environmental benefits across multiple clinical areas. By framing prescribing decisions through the dual lens of sustainability and value-based healthcare, projects like this can contribute meaningfully to both patient care and planetary health.

Limitations of the study

This project has several limitations that should be acknowledged. Firstly, to capture a broad and representative dataset, we opted to include all 1 g doses of oral and IV paracetamol dispensed to the ED during each audit cycle. We did not use any exclusion criteria for patient groups who may have a clear clinical indication for IV administration. These include patients who were nil by mouth due to swallowing difficulties or had severe nausea/vomiting; patients awaiting surgical review; patients with reduced consciousness; and other similar clinical situations where oral administration would not have been appropriate. By excluding these patients, it is likely that the overall proportion of IV use would have been lower.

We acknowledge that applying exclusion criteria could have provided a more accurate representation of unnecessary IV prescribing. However, this would have required a long list of criteria alongside subjective, case-by-case analysis. Instead, by capturing all issued doses, we believe the overall picture already accounts for these factors and provides a pragmatic reflection of prescribing behaviour at the departmental level. Future cycles could be strengthened by incorporating patient-level chart reviews to help distinguish clinically justified IV use from potentially avoidable prescribing, thereby providing greater precision in estimating the true scope for improvement.

A second limitation is that the data were derived from pharmacy issue records rather than direct prescribing or administration records. Although pharmacy records provide a reliable measure of the volume of paracetamol dispensed to the ED, they do not account for wastage or dose omissions. Nonetheless, given the large number of doses and the extended timeframe of data collection, we believe these factors are unlikely to have significantly altered the overall trends observed.

Finally, as with many QI initiatives, the generalisability of our findings may be limited by the specific practices of our department, including local prescribing habits, staff skillsets, and workflow processes. The single-centre design further constrains wider applicability. However, the methodology of using structured interventions and iterative PDSA cycles remains widely transferable to other contexts, and the principle of optimising oral over IV prescribing retains broad clinical, financial, and environmental relevance.

Alongside these limitations, several strengths should also be noted. This project drew on a large dataset collected over multiple audit cycles and across an extended observation period, which provides robustness to the findings. The structured application of QI methodology, including the use of process mapping, driver diagrams, and a SMART aim, enhanced the project’s strength compared to informal approaches. Furthermore, framing prescribing practices in terms of both financial and environmental impact adds a valuable dimension that extends the project’s relevance beyond the local setting.

Evidence from the literature

Cost and Clinical Effectiveness

A 2020 systematic review of perioperative trials found that the route of paracetamol administration (IV vs. oral) did not meaningfully impact postoperative pain outcomes or secondary measures such as opioid consumption, patient satisfaction, or length of hospital stay [5]. Across 14 randomised controlled trials including 1,695 participants, pain scores were comparable between groups at all assessed time intervals. Although IV formulations achieve higher plasma concentrations more rapidly, this did not translate into superior clinical outcomes, reinforcing the view that oral administration is appropriate in most cases where feasible [5].

Building on this, the same review highlighted the substantial cost difference between formulations, with an IV dose reported to be approximately 10 times more expensive than an equivalent oral dose (£1.95 vs. £0.19). At Erasmus Medical Center in the Netherlands, annual expenditure on IV paracetamol was estimated at £85,910. Modelling from the same review suggested that replacing just half of these IV administrations with oral paracetamol would save approximately £38,711 per year without compromising clinical outcomes [5]. Extrapolated across multiple departments or entire healthcare systems, such figures underscore the considerable financial benefit of optimising prescribing practices to favour the oral route whenever clinically appropriate.

Further evidence from real-world practice reinforces these findings. A retrospective study of 579 patients undergoing laparoscopic cholecystectomy compared a single preoperative oral dose of paracetamol with IV administration. The analysis found no meaningful difference in postoperative pain control, with the median pain score in the post-anaesthesia care Unit (PACU) being a two out of 10 in both groups. This indicates that patients reported only mild pain on average, regardless of the route of administration. Opioid consumption and PACU length of stay were likewise similar between groups. Importantly, the oral route avoided the added cost of IV preparation and administration, highlighting that clinical outcomes were maintained while achieving significant financial savings [6].

Environmental Impact

The NHS has made a statutory commitment to achieve net-zero carbon emissions by 2040 for the emissions it controls directly (the NHS Carbon Footprint) and by 2045 for those it can influence indirectly (the NHS Carbon Footprint Plus) [7]. This project contributes to those goals by targeting a small but meaningful reduction in pharmaceutical-related greenhouse gas emissions through route optimisation.

CO₂e is a standard unit used to compare the climate impact of different greenhouse gases by expressing them in terms of the amount of CO₂ that would have the same warming effect. Life-cycle assessments of paracetamol have shown that a 1 g oral tablet generates approximately 38 g of CO₂e, whereas a 1 g IV dose emits 310-628 g of CO₂e, giving it up to a 16-fold higher carbon footprint [2]. To put this into perspective, giving one IV dose instead of an oral tablet is equivalent to driving a petrol car for one to two miles. When multiplied across various departments and hospital systems, such reductions not only contribute directly to the NHS's net-zero pathway but also exemplify how incremental prescribing changes can support the NHS’s ambition to become the world’s first net-zero national health service. 

Why Route Matters?

The choice of drug administration route has important implications not only for clinical care but also for sustainability. IV therapy generally requires more resources than oral administration, including consumables such as cannulas, infusion sets, syringes, and sterile packaging, all of which contribute to higher waste generation and energy use. An eco-audit conducted in a French university hospital highlighted that the IV administration of paracetamol and ketoprofen generated 444-556 g of CO₂e and 9.8 to 12.2 litres of water waste, in comparison to oral administration, which produced only 8.36 g of CO₂e and consumed 1.16 L of water, a difference of more than 50-fold in carbon emissions and water use [8]. 

While IV paracetamol may sometimes be clinically indicated, its administration inevitably requires insertion of an IV cannula and the use of other single-use plastics such as syringes, needles, and sterile packaging. These consumables carry their own environmental costs and compound the footprint of the drug itself. Evidence from clinical practice illustrates this impact clearly. At Charing Cross Hospital in London, a baseline audit showed that 86% of ED patients were cannulated, yet only 40% of those cannulas were actually used. Recognising both the financial and environmental burden of this practice, an education and awareness project aimed at reducing unnecessary cannulation was launched. By raising staff awareness of the costs and climate impact of unused IV lines, the project achieved a 25% reduction in cannulation rates, corresponding to an estimated 19,000 kg reduction in CO₂ emissions per year and savings of approximately £95,000 [9]. 

The broader pharmaceutical literature reinforces that the route and packaging of administration often outweigh the intrinsic environmental cost of the active ingredient itself. A life-cycle assessment of morphine production found that the final stages of sterilisation and single-use packaging were responsible for nearly 90% of its total carbon footprint, even though the earlier steps of poppy cultivation and chemical synthesis might have seemed more resource-intensive. This highlights that the activities closest to the point of care, such as preparing, packaging, and delivering the drug, can have a far greater environmental impact than the bulk manufacturing processes themselves [10]. This mirrors the findings from a Swedish ICU, where around 63% of the carbon footprint per inpatient day was attributable to single-use items such as syringes and aprons [11]. Together, these studies demonstrate that the materials and processes associated with IV administration significantly drive emissions. When applied to paracetamol, they underscore that the true cost of IV therapy extends well beyond the drug itself, encompassing the hidden environmental burden of its delivery system. 

From a patient‑safety perspective, IV administration is markedly more error‑prone than oral routes. A systematic review and Bayesian analysis estimated that the overall probability of making at least one error during IV therapy is approximately 73%, with the reconstitution step being the most error-prone. Introducing error checks at every stage reduced the probability to 22%, and using pre-prepared injections lowered it further to 17%. These findings demonstrate the complexity of IV administration and highlight how small process changes can significantly reduce risk [12]. 

A UK-based review further reinforced this disparity, illustrating that IV doses were around five times more likely to result in a medication error compared to non-IV doses. Reported error rates were 35% for IV medications, in contrast to just 6% for oral or other non-IV routes [13]. 

Direct observational studies add further insight into the frequency and severity of these errors. In a study of 568 IV administrations across two hospitals, almost 70% involved at least one clinical error, with one in four being classified as serious. Four types of error accounted for over 90% of all cases: incorrect administration rate, incorrect mixture, incorrect volume, and drug incompatibility. Risk was higher when medications were given as a bolus but decreased with increased clinical experience [14]. 

Taken together, these findings demonstrate that IV medications carry a significantly higher risk of error and patient harm compared to oral drugs. Promoting oral paracetamol when clinically appropriate is therefore not only a cost-effective and environmentally sustainable approach but also a safer option for patients.

Similar QI Initiatives 

Across the world, several QI and sustainability initiatives have targeted the overuse of IV paracetamol, highlighting that relatively small prescribing changes can yield large financial and environmental benefits without compromising patient care. These projects provide a valuable benchmark for the present study. 

Hunfeld and colleagues at Erasmus University Medical Center launched the “Paracetamol Challenge” after data showed that nearly half (49%) of all ICU paracetamol administrations in early 2023 were IV [15]. Through posters and an awareness campaign, staff were encouraged to reduce IV use by 25%. Within a year, the average number of IV paracetamol doses per patient fell by almost half (from 10.2 to 5.2), equating to 730 fewer IV doses in a single month. This translated to 106 kg less waste, 1,380 euros in savings, and a reduction of 458 kg of CO₂ emissions. Over 40 hospitals across the Netherlands adopted the initiative, highlighting its scalability [15]. This mirrors our own project, showing how simple interventions around the route of administration can deliver meaningful environmental and financial benefits. 

Similarly, a U.S. academic medical centre implemented a multi-cycle QI project using PDSA methodology to reduce inappropriate IV paracetamol use [16]. Interventions included refining restriction criteria, embedding clinical decision support into the electronic medical record, educating staff on appropriate use, and empowering pharmacists to enforce prescribing rules. As a result, monthly spending on IV paracetamol dropped from over $56,000 to just $5,800. Importantly, this was achieved without any increase in opioid or alternative analgesic use, confirming that IV paracetamol can be safely restricted in a cost-effective manner [16]. 

At Grange University Hospital in the UK, a sustainability project was introduced in the ICU in 2023 to reduce unnecessary IV paracetamol prescribing [17]. In collaboration with the pharmacy team, prompts were added to automated dispensing units. The prompts encouraged nursing staff to consider oral administration when clinically appropriate. This resulted in a 21% reduction in IV use, with fewer doses given without a clear indication. The project demonstrated reductions in cost and plastic waste and was later expanded across inpatient wards and the wider health board [17]. 

Together, these projects demonstrate that targeted interventions to reduce unnecessary IV paracetamol use can reliably deliver meaningful financial savings, lower environmental impact, and maintain patient safety. By situating our work within this wider context, our initiative aims to reinforce the evidence that route optimisation is a practical and scalable strategy. The findings from other initiatives not only validate the importance of our local efforts at Royal Preston Hospital but also highlight the potential for similar projects to be replicated across EDs and healthcare systems more broadly.

Future directions

This QI project illustrates how routine prescribing practices can be optimised to potentially deliver gains in patient safety, financial efficiency, and environmental sustainability. Although informal interventions such as posters and word-of-mouth teaching were well-intentioned, their limited impact reinforces the need for structured QI projects that incorporate regular feedback and targeted behavioural strategies. 

One promising avenue for future work lies in the integration of electronic prescribing systems with embedded clinical decision support. A narrative review in 2014 concluded that the combination of computerised provider order entry and clinical decision support systems consistently reduces prescribing errors, although the impact on clinical adverse drug events is less reliable due to alert fatigue and other implementation challenges [18]. Nevertheless, these findings suggest that tailored decision support prompts could reinforce safe and sustainable prescribing choices, such as favouring the oral route of paracetamol when clinically appropriate. 

In parallel, educational reform is essential to embed sustainable prescribing practices into everyday clinical behaviour. The General Medical Council UK has formally incorporated sustainable healthcare into its outcomes for graduates, requiring that new doctors understand and apply principles of environmentally responsible care [19]. However, a 2022 survey of UK medical students highlighted a major gap in training. While 93% of participants agreed that climate change is an important issue, only 1.8% reported having been formally taught about sustainable healthcare, and just 3.1% felt confident answering exam questions on the topic. Notably, 89% expressed a clear demand for more teaching, with most preferring it to be integrated across both preclinical and clinical years [20]. These findings emphasise that while the mandate for sustainable healthcare education is well established, implementation remains inconsistent, leaving projects such as this well placed to serve as practical learning opportunities that bridge the gap between policy and practice. By aligning local QI projects with national net-zero ambitions, this initiative demonstrates how everyday prescribing decisions can be reframed as opportunities for safer, greener, and more sustainable healthcare delivery. 

## Conclusions

This project demonstrates how structured QI methodology can be applied to optimise paracetamol prescribing in the ED. Our findings suggested that informal, ad hoc interventions did not deliver lasting change, whereas adopting a systematic, multi-cycle approach offers the potential for sustained improvement. By linking prescribing behaviour to cost, patient safety, and environmental sustainability, this initiative highlights how even small changes in practice can have wide-reaching benefits. The primary aim of this article was not only to share our experience but also to encourage other departments, hospitals, and health systems to replicate similar QI-driven projects. Through collective effort, route optimisation of common medications such as paracetamol can contribute to safer, greener, and more cost-effective healthcare on a global scale.
